# Laboratory Investigation and Phylogenetic Analysis of an Imported Middle East Respiratory Syndrome Coronavirus Case in Greece

**DOI:** 10.1371/journal.pone.0125809

**Published:** 2015-04-28

**Authors:** Athanasios Kossyvakis, Ying Tao, Xiaoyan Lu, Vasiliki Pogka, Sotirios Tsiodras, Mary Emmanouil, Andreas F. Mentis, Suxiang Tong, Dean D. Erdman, Antonios Antoniadis

**Affiliations:** 1 National Influenza Reference Laboratory of Southern Greece, Hellenic Pasteur Institute, Athens, Greece; 2 Division of Viral Diseases, Centers for Disease Control and Prevention, Atlanta, Georgia, United States of America; 3 Department of Epidemiological Surveillance and Intervention, Hellenic Centre for Disease Control and Prevention, Athens, Greece; University of Texas Medical Branch, UNITED STATES

## Abstract

Rapid and reliable laboratory diagnosis of persons suspected of Middle East respiratory syndrome coronavirus (MERS-CoV) infection is important for timely implementation of infection control practices and disease management. In addition, monitoring molecular changes in the virus can help elucidate chains of transmission and identify mutations that might influence virus transmission efficiency. This was illustrated by a recent laboratory investigation we conducted on an imported MERS-CoV case in Greece. Two oropharyngeal swab specimens were collected on the 1^st^ and 2^nd^ day of patient hospitalization and tested using two real-time RT-PCR (rRT-PCR) assays targeting the UpE and Orf-1a regions of the MERS-CoV genome and RT-PCR and partial sequencing of RNA-dependent RNA polymerase and nucleocapsid genes. Serum specimens were also collected and serological test were performed. Results from the first swab sample were inconclusive while the second swab was strongly positive for MERS-CoV RNA by rRT-PCR and confirmed positive by RT-PCR and partial gene sequencing. Positive serologic test results further confirmed MERS-CoV infection. Full-length nucleocapsid and spike gene coding sequences were later obtained from the positive swab sample. Phylogenetic analysis revealed that the virus was closely related to recent human-derived MERS-CoV strains obtained in Jeddah and Makkah, Saudi Arabia, in April 2014 and dromedary camels in Saudi Arabia and Qatar. These findings were consistent with the patient’s history. We also identified a unique amino acid substitution in the spike receptor binding domain that may have implications for receptor binding efficiency. Our initial inconclusive rRT-PCR results highlight the importance of collecting multiple specimens from suspect MERS-CoV cases and particularly specimens from the lower respiratory tract.

## Introduction

Since its discovery in Saudi Arabia in 2012, the Middle East respiratory syndrome coronavirus (MERSCoV) has been implicated in 1042 laboratory confirmed cases of human infection, including 419 deaths. Among these cases, 14 were reported by European countries, including 8 imported cases and 3 local person-to-person transmissions [1].

The MERS-CoV genome is comprised of at least 10 putative open reading frames, including 2 that encode the spike (S) and nucleocapsid (N) proteins [2]. The S and N protein genes have been used for coronavirus genotyping and phylogenetic analyses which have aided our understanding of the virus’s temporal and geographic origins and evolution [3]. The S protein is involved in virus receptor binding and is known to be the main antigenic component to which significant neutralizing antibody responses are induced [4]. The N protein is a highly immunogenic phosphoprotein implicated in viral genome replication and modulation of cell signaling pathways [5].

The present study focused on the laboratory investigation of an imported MERS-CoV case in Greece and its phylogenetic comparison with other recently circulating strains.

## Materials and Methods

A 69-year-old Greek national presented to a tertiary care hospital in Athens on 17 April 2014 with prolonged fever, diarrhea and pneumonia (onset of symptoms on 8 April). He had arrived a few hours earlier from Jeddah, Saudi Arabia, where he resides permanently [6]. Because of high suspicion of MERS-CoV infection, two oropharyngeal swab specimens were collected on 17 April (specimen A) and 18 April (specimen B) for laboratory investigation. The purpose of the urgent clinical care testing needed was explained to the patient and written consent was obtained for potential publication.

For molecular detection of MERS-CoV RNA, two real-time RT-PCR (rRT-PCR) assays targeting regions upstream of envelope gene (UpE) and the open reading frame (ORF) 1a were used [7]. Partial sequencing of the RNA-dependent RNA polymerase (RdRp) and N genes and BLAST analysis of the amplicon sequences was also performed as previously described [7]. A serum sample collected from the patient after six days of hospitalization was available for serological testing using the Anti-MERS Coronavirus Indirect Immunofluoresence kit (Euroimmun, Lubeck, Germany).

In addition to partial sequencing, full length ORFs of the S (4042 nt) and N (1242 nt) protein genes were obtained by the Centers for Disease Control and Prevention (CDC), Atlanta, GA, using Sanger sequencing methods and deposited into the GenBank database under accession numbers KJ782550 and KJ782549, respectively. Sequences were confirmed independently by two separate CDC laboratories.

## Results

The UpE and ORF-1a rRT-PCR results for specimen A were equivocal [PCR threshold cycle (Ct) value 38.8] and negative, respectively, and the specimen was determined to be inconclusive for MERS-CoV RNA detection. In contrast, specimen B was found to be positive by both UpE and ORF-1a assays with Ct values 30.2 and 32.5, respectively. The presence of MERS-CoV RNA was also confirmed on specimen B by RT-PCR and partial sequencing of the RdRp and N genes.

Full genome sequencing was not possible due to the limited available sample volume. However, sequences of the S and N protein coding regions were successfully obtained. The *Greece-Saudi Arabia_2014* S differed by 0.1–0.9% nucleotides (nt) and 0.1–1.3% amino acids (aa) from 68 other published human and camel derived MERS-CoV sequences. One unique nt change present as a mixed base at position 1533 (G>C) of the S ORF confers a predicted aa change of arginine (R) with proline (P) at position 511 (R511P). The *Greece-Saudi Arabia_2014* N differed by 0.1–0.9% nt and 0–0.8% aa from 69 other published MERS-CoV sequences. No unique nt changes were identified.

**Fig 1:**
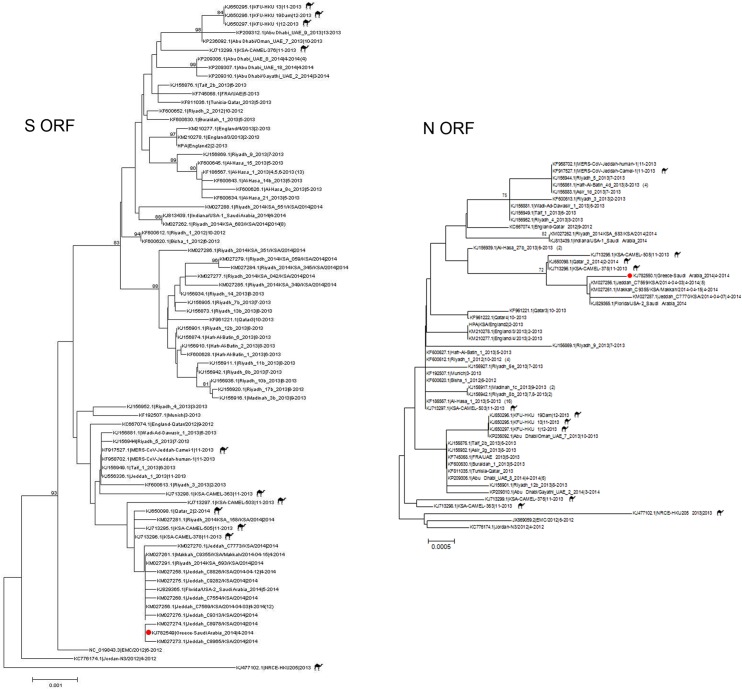
Phylogenetic analyses of the MERS-CoV S and N ORFs. Midpoint-rooted phylogenetic trees of the full-length nucleocapsid (N) and spike (S) open-reading frames (ORFs) obtained from the clinical sample and published nucleotide sequences available from i) GenBank, ii) the Health Protection Agency (HPA) website (http://www.hpa.org.uk/webw/HPAweb&HPAwebStandard/HPAweb_C/1317136246479) and iii) the Institut Fr Virologie (IFV) website (http://www.virology-bonn.de/index.php?id=46). The estimated neighbor-joining trees were constructed from nucleotide alignments using MEGA version 6.06. Sequence names are derived from Genbank accession number|virus strain name|month-year of collection. Numbers in the parentheses denote additional human sequences identical to the listed sequence. An asterisk (*) denotes 2 additional identical N ORF sequences obtained from IFV, including strains MERS-CoV/Jeddah_2014_C8826 and MERS-CoV/Jeddah_2014_C9055. Two asterisks (**) denote 3 additional identical S ORF sequences obtained from IFV, including MERS-CoV/Jeddah_2014_C9055, MERS-CoV/Jeddah_2014_C7770 and MERS-CoV/Jeddah_2014_C7149. MERS-CoV sequences derived from camel specimens indicated by camel icon. Bootstrap support values (1000 replicates) 75% are plotted at the indicated internal branch nodes. Scale bars show the number of nucleotide substitutions per site.

Phylogenetic analyses of the MERS-CoV S and N ORFs are shown in Fig 1. The *Greece-Saudi Arabia_2014* sequences clustered most closely with human derived MERS-CoV strains obtained in Jeddah and Makkah, Saudi Arabia, in April 2014. MERS-CoV sequences recently obtained from dromedary camels in Saudi Arabia and Qatar also clustered with the *Greece-Saudi Arabia_2014* sequences.

Serology identified specific anti-MER-CoV IgM (titer 1/100) and IgG (titer 1/320) antibodies in the patient’s serum sample.

## Discussion

The present study describes the laboratory investigation of an imported MERS-CoV case in Greece. Discrepancy of the rRT-PCR results between specimens A and B may be due to different sampling techniques, although detection attributable to increasing viral load with infection progression cannot be ruled out for specimen B. Collection of additional specimens, particularly from the lower respiratory tract and at different time points over the disease course is highly recommended when there is strong clinical and epidemiological suspicion of MERS-CoV infection and initial results from upper respiratory tract specimens are negative or inconclusive. Drosten et al. pointed out that up to 10^6^ copies/mL of MERS-CoV RNA were present in lower respiratory tract specimens, whereas only small amounts of viral RNAs could be detected in upper respiratory tract specimens [8].

According to World Health Organization recommendations, partial sequencing of the RdRp and N genes is an acceptable alternative for confirmation of MERS-CoV infection [7]. Although RT-PCR and partial gene sequencing methods are more time consuming, we found this approach to be very helpful in resolving our discrepant RT-PCR results.

Detection of MERS-CoV-specific antibodies in the patient’s serum was consistent with recent MERS-CoV infection. Ideally, however, for serological confirmation of acute MERS-CoV infection, a second serum specimen taken two weeks or more after onset of illness should also be tested.

Our findings revealed a genomic subpopulation in the swab sample that possessed a unique amino acid substitution in S protein receptor-binding domain (RBD) as compared with other published human and camel sequences. The above substitution was confirmed independently by two separate CDC laboratories, ruling out the possibility of its introduction during the RT-PCR. The R511P substitution is located within a region (residues 484 to 567) of the MERS-CoV RBD that directly interacts with the dipeptidyl peptidase4 (DPP4) receptor on the host cell surface for viral attachment [9–12]. Recent mutagenesis studies indicated that alterations of key residues within that region, i.e., the D510A and the E513A, have been shown to significantly reduce both binding and viral entry efficiency [9, 13]. The same studies indicated that the R511A substitution had little to no effect to the binding affinities of the RBD for DPP4. Further studies are needed to clarify whether the substitution of arginine to proline in the same position (R511P) identified in the present study had any functional implication.

Phylogenetic analysis revealed that the Greek strain was most closely related to Jeddah and Makkah strains circulating during the recent burst of MERS-CoV activity in the Arabian Peninsula [14]. This was consistent with the assumption that the patient might have acquired the infection in the community or, alternatively, in a nosocomial-environment, as he had visited a local hospital in Jeddah repeatedly since the beginning of April 2014. Moreover, the clustering of the Greek S and N ORFs with sequences from some dromedary camels was in accordance with recent findings, confirming the similarity between human and camel MERS-CoV sequences identified in the same geographical areas [15, 16].

The ongoing outbreak of MERS-CoV suggests that the risk of importation of the virus in countries is expected to continue, although the number of incident cases has been steadily declining [1]. In that respect, fast and reliable laboratory confirmation is crucial for timely implementation of infection control measures to prevent secondary person to person transmission.

## References

[1] European Center for Disease Control. Severe respiratory disease associated with Middle East respiratory syndrome coronavirus. Available: http://www.ecdc.europa.eu/en/publications/Publications/MERS_update_14-Feb2014.pdf. Accessed 23 February 2015.

[2] Boheemen S, Graaf M, Lauber C, Bestebroer TM, Raj VS, Zaki AM, et al Genomic Characterization of a Newly Discovered Coronavirus Associated with Acute Respiratory Distress Syndrome in Humans. mBio. 2012;3(6). 10.1128/mBio.00473-12 PMC3509437 23170002

[3] Cotten M, Lam TT, Watson SJ, Palser AL, Petrova V, Grant P, et al Full-genome deep sequencing and phylogenetic analysis of novel human betacoronavirus. Emerg Infect Dis. 2013 5;19(5):736–42. 10.3201/eid1905.130057 23693015 PMC3647518

[4] Qian Z, Dominguez SR, Holmes KV. Role of the spike glycoprotein of human Middle East respiratory syndrome coronavirus (MERS-CoV) in virus entry and syncytia formation. PLoS One. 2013;8(10):e76469 10.1371/journal.pone.0076469 24098509 PMC3789674

[5] Jayaram H, Fan H, Bowman BR, Ooi A, Jayaram J, Collisson EW, et al X-ray structures of the N- and C-terminal domains of a coronavirus nucleocapsid protein: implications for nucleocapsid formation. J Virol. 2006 7;80(13):6612–20. 16775348 10.1128/JVI.00157-06 PMC1488953

[6] Tsiodras S, Baka A, Mentis A, Iliopoulos D, Dedoukou X, Papamavrou G, et al A case of imported Middle East Respiratory Syndrome coronavirus infection and public health response, Greece, April 2014. Euro Surveill. 2014;19(16). 24786258 10.2807/1560-7917.es2014.19.16.20782

[7] Corman VM, Muller MA, Costabel U, Timm J, Binger T, Meyer B, et al Assays for laboratory confirmation of novel human coronavirus (hCoV-EMC) infections. Euro Surveill. 2012;17(49). 23231891 10.2807/ese.17.49.20334-en

[8] Drosten C, Seilmaier M, Corman VM, Hartmann W, Scheible G, Sack S, et al Clinical features and virological analysis of a case of Middle East respiratory syndrome coronavirus infection. Lancet Infect Dis. 2013 9;13(9):745–51. 10.1016/S1473-3099(13)70154-3 23782859 PMC7164791

[9] Wang N, Shi X, Jiang L, Zhang S, Wang D, Tong P, et al Structure of MERS-CoV spike receptor-binding domain complexed with human receptor DPP4. Cell Res. 2013 8;23(8):986–93. 10.1038/cr.2013.92 23835475 PMC3731569

[10] Chen Y, Rajashankar KR, Yang Y, Agnihothram SS, Liu C, Lin YL, et al Crystal structure of the receptor-binding domain from newly emerged Middle East respiratory syndrome coronavirus. J Virol. 2013 10;87(19):10777–83. 10.1128/JVI.01756-13 23903833 PMC3807420

[11] Raj VS, Mou H, Smits SL, Dekkers DH, Muller MA, Dijkman R, et al Dipeptidyl peptidase 4 is a functional receptor for the emerging human coronavirus-EMC. Nature. 2013 3 14;495(7440):251–4. 10.1038/nature12005 23486063 PMC7095326

[12] Lu L, Liu Q, Zhu Y, Chan K, Qin L, Li Y, et al Structure-based discovery of Middle East respiratory syndrome coronavirus fusion inhibitor. Nat Commun. 2014;5. 10.1038/ncomms4067 PMC7091805 24473083

[13] Du L, Zhao G, Yang Y, Qiu H, Wang L, Kou Z, et al A conformation-dependent neutralizing monoclonal antibody specifically targeting receptor-binding domain in Middle East respiratory syndrome coronavirus spike protein. J Virol. 2014 6;88(12):7045–53. 10.1128/JVI.00433-14 24719424 PMC4054355

[14] European Centre for Disease Prevention and Control. Epidemiological update: Middle East respiratory syndrome coronavirus (MERS-CoV). Available: http://www.ecdc.europa.eu/en/press/news/_layouts/forms/News_DispForm.aspx?List=8db7286c-fe2d-476c-9133-18ff4cb1b568&ID=998. Accessed 06 May 2014.

[15] Briese T, Mishra N, Jain K, Zalmout SI, Jabado JO, Karesh BW, et al Middle East Respiratory Syndrome Coronavirus Quasispecies That Include Homologues of Human Isolates Revealed through Whole-Genome Analysis and Virus Cultured from Dromedary Camels in Saudi Arabia. mBio. 2014;5(3). 10.1128/mBio.01146-14 PMC4010836 24781747

[16] Chu DK, Poon LL, Gomaa MM, Shehata MM, Perera RA, Abu Zeid D, et al MERS coronaviruses in dromedary camels, Egypt. Emerg Infect Dis. 2014 6;20(6):1049–53. 10.3201/eid2006.140299 24856660 PMC4036765

